# Supplementary figures and images for: Unsupervised learning analysis on the proteomes of Zika virus
Source: PeerJ Comput Sci. 2024 Nov 11;10:e2443. doi: 10.7717/peerj-cs.2443 (PMC11623125; doi:10.7717/peerj-cs.2443)

ntree

|     |  |      |  |      |  |
|-----|--|------|--|------|--|
| 250 |  | 1000 |  | 2000 |  |
| 500 |  | 1500 |  |      |  |

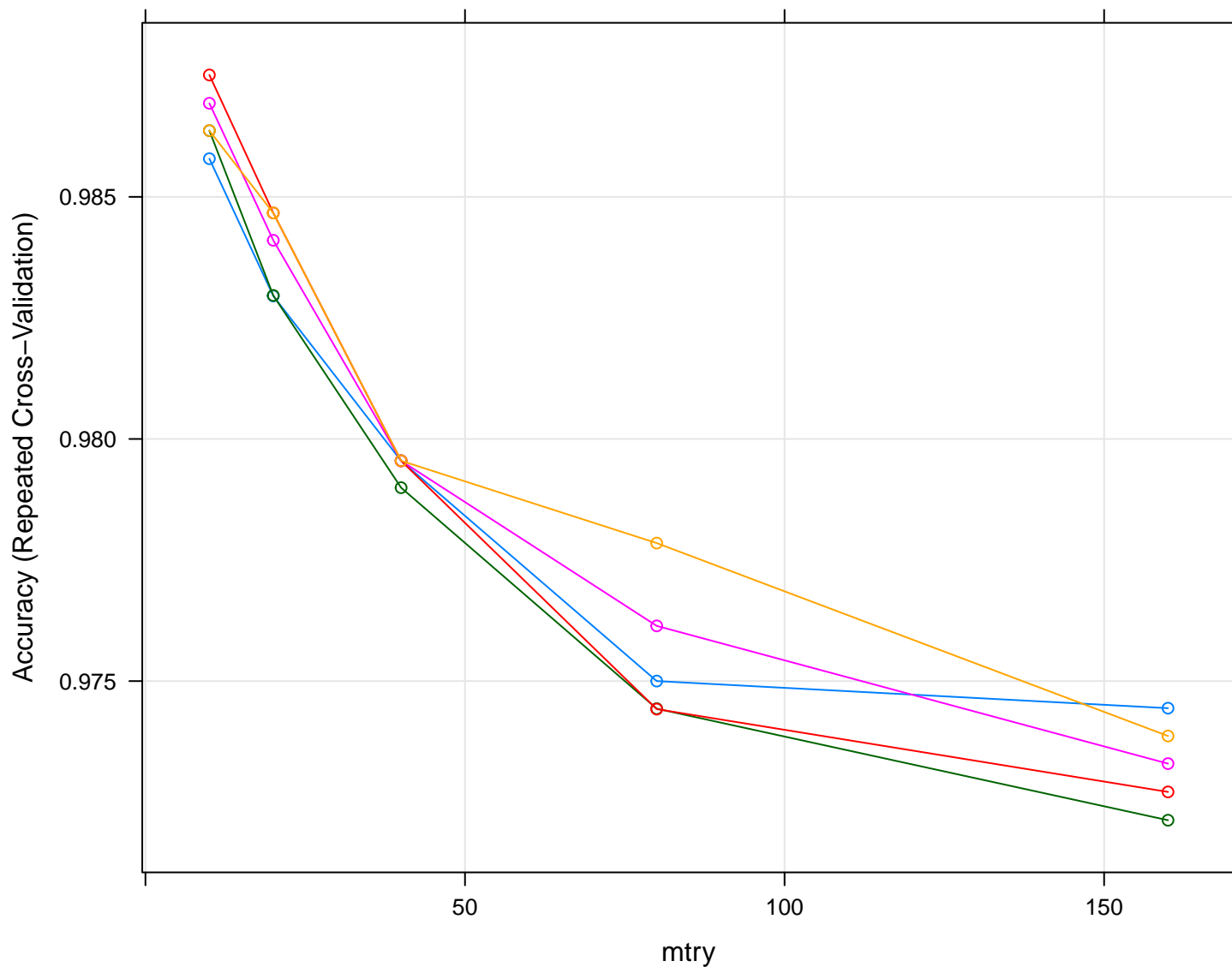

Supplement: Figure S1 — The mtry parameter is shown in the x-axis and the accuracy performance in the y-axis for each model using different number of trees (colored lines). [file peerj-cs-10-2443-s008.pdf]

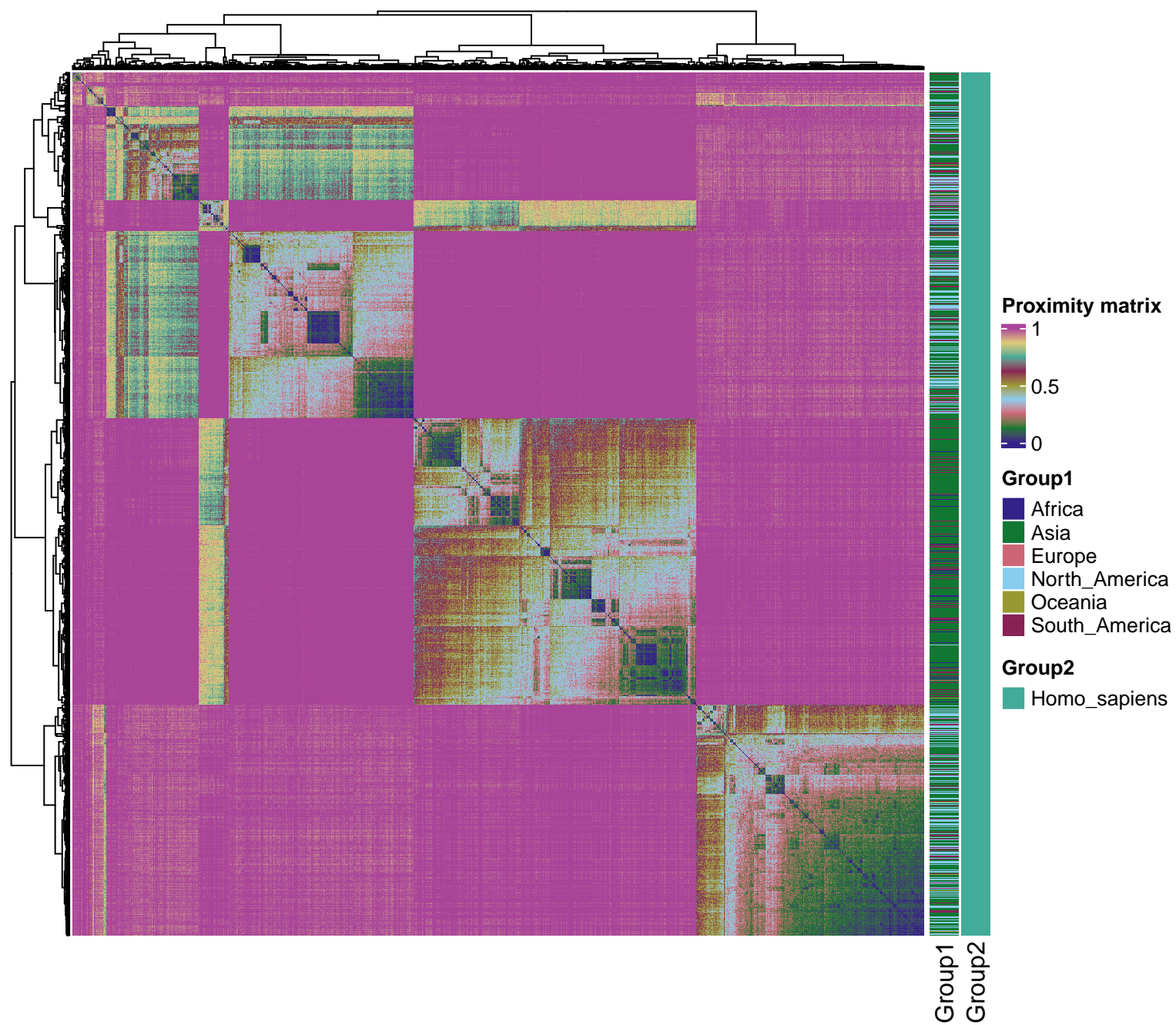

Supplement: Figure S2 — The right bar colors indicate the groups by geographic regions (group 1) and human host (group 2). The muted nine-style colorblind-friendly (https://github.com/JLSteenwyk/ggpubfigs) in the blocks indicates the similarity within the groups. [file peerj-cs-10-2443-s009.pdf]

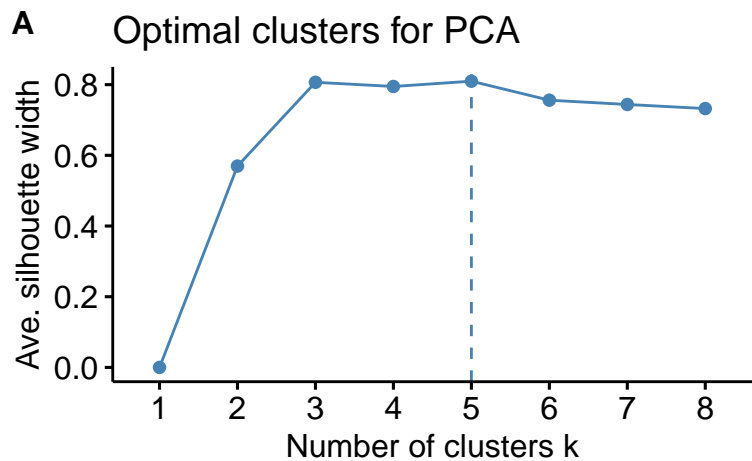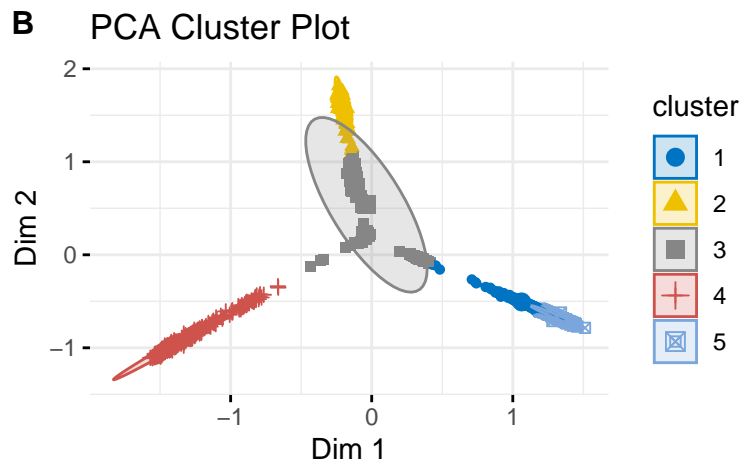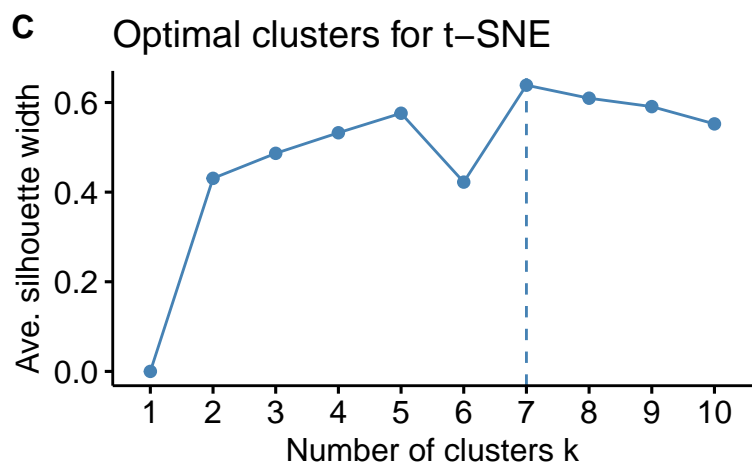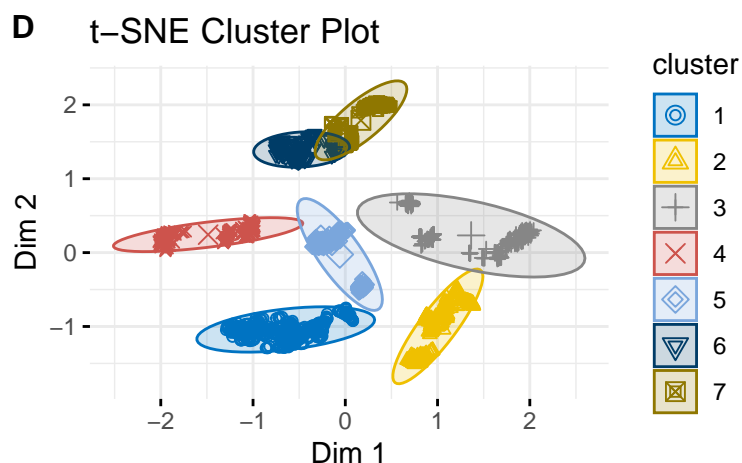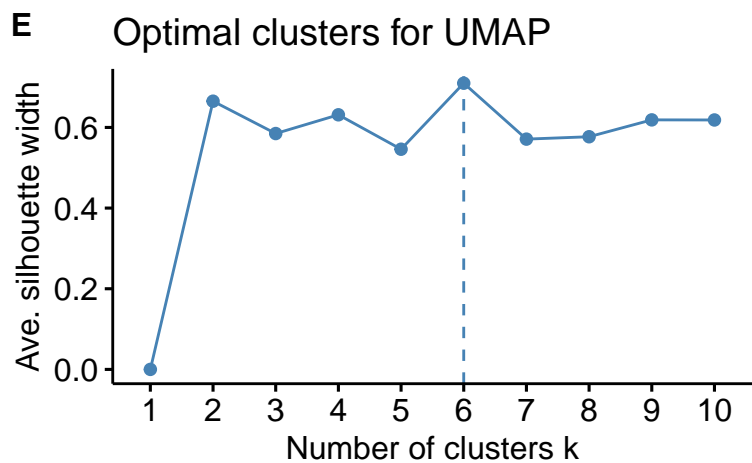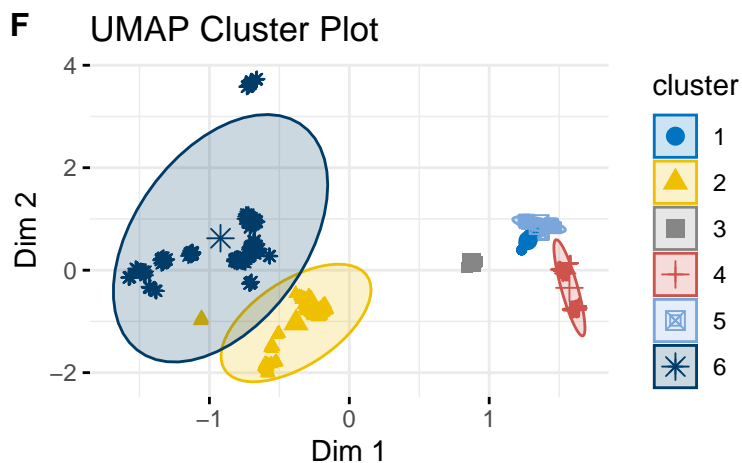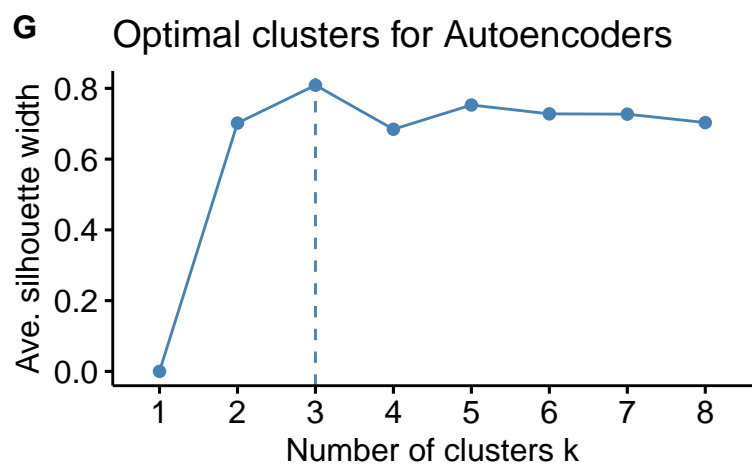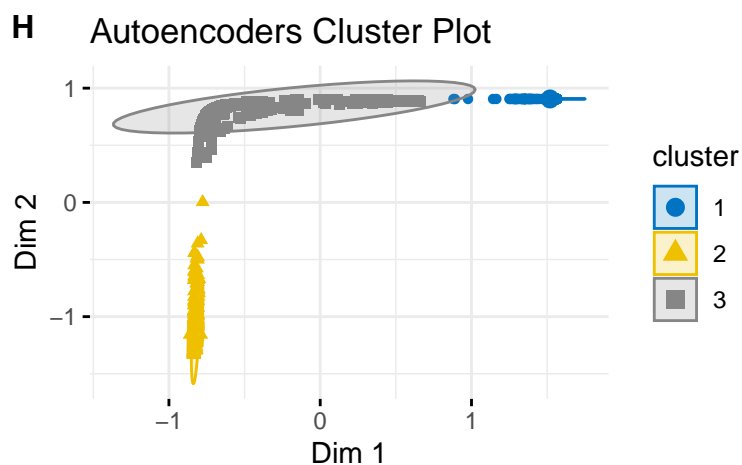

Supplement: Figure S3 — The elbow curves (A, C, E, G) and cluster (B, D, F, H) plots based on k means clustering using Euclidean distance. [file peerj-cs-10-2443-s010.pdf]
